# Parathyroid Hormone: A Uremic Toxin

**DOI:** 10.3390/toxins12030189

**Published:** 2020-03-17

**Authors:** Eduardo J. Duque, Rosilene M. Elias, Rosa M. A. Moysés

**Affiliations:** 1 LIM 16, Nephrology Department, Hospital das Clínicas HCFMUSP, Universidade de São Paulo, São Paulo 05403-000, Brazil; eduardojorgeduque@gmail.com (E.J.D.); rosilenemotta@hotmail.com (R.M.E.); 2 Post-Graduation, Universidade Nove de Julho (UNINOVE), São Paulo 01525-000, Brazil

**Keywords:** parathyroid hormone, secondary hyperparathyroidism, uremic toxin

## Abstract

Parathyroid hormone (PTH) has an important role in the maintenance of serum calcium levels. It activates renal 1α-hydroxylase and increases the synthesis of the active form of vitamin D (1,25[OH]_2_D_3_). PTH promotes calcium release from the bone and enhances tubular calcium resorption through direct action on these sites. Hallmarks of secondary hyperparathyroidism associated with chronic kidney disease (CKD) include increase in serum fibroblast growth factor 23 (FGF-23), reduction in renal 1,25[OH]_2_D_3_ production with a decline in its serum levels, decrease in intestinal calcium absorption, and, at later stages, hyperphosphatemia and high levels of PTH. In this paper, we aim to critically discuss severe CKD-related hyperparathyroidism, in which PTH, through calcium-dependent and -independent mechanisms, leads to harmful effects and manifestations of the uremic syndrome, such as bone loss, skin and soft tissue calcification, cardiomyopathy, immunodeficiency, impairment of erythropoiesis, increase of energy expenditure, and muscle weakness.

## 1. Introduction

Parathyroid hormone (PTH) is a 9400 D molecular weight peptide, containing 84 amino acids that are secreted after cleavage from preproparathyroid hormone (115 amino acids) to proparathyroid hormone (90 amino acids). The active biological form is the intact PTH (1–84), whose half-life in the circulation is less than three minutes, and which clearance occurs mainly in the liver (60%–70%) and kidney (20%–30%) [1].

The secretion of PTH is regulated by changes of extracellular calcium through a feedback mainly mediated by the calcium-sensing receptor (CaSR). This receptor, a G protein-coupled receptor on parathyroid cells, regulates calcium-influenced PTH secretion [2]. A reduction of ionized calcium stimulates the PTH secretion, whereas high levels suppress the PTH release and enhance calcitonin secretion.

**Figure 1:**
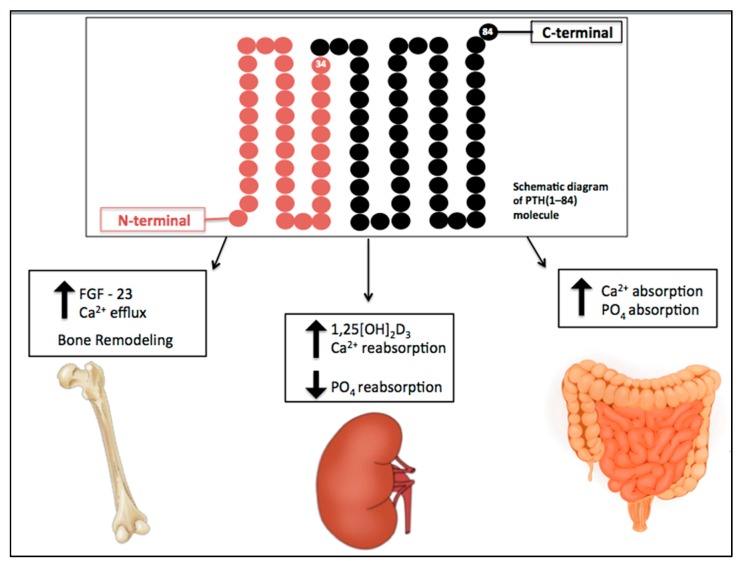
Physiological actions of parathyroid hormone (PTH). PTH plays a key role in the maintenance of calcium levels. It stimulates bone turnover and calcium release from the skeleton. In renal tubular cells, PTH increases calcium reabsorption, inhibits phosphate reabsorption, and upregulates the 1α-hydroxylase gene, responsible for conversion of 25-hydroxyvitamin D to the active metabolite, 1,25[OH]_2_D_3_. It also enhances calcium and phosphate intestinal absorption by increasing the production of activated vitamin D. Down arrow = decrease, Up arrow = increase.

The effects of PTH are summarized in Figure 1. In renal proximal tubular cells, PTH inhibits phosphate reabsorption and upregulates the 1α-hydroxylase gene, responsible for conversion of 25-hydroxyvitamin D to the active metabolite 1,25-dihydroxyvitamin D (1,25[OH]_2_D_3_). It also increases calcium reabsorption by inserting calcium channels in the apical membrane of distal tubules and stimulating basolateral sodium-calcium transporters [3].

In bone tissue, PTH influences gene expression in osteoblasts, supporting the synthesis of proteins required for bone formation and osteoclast differentiation. Intermittent exposure to PTH is antiosteoporotic and osteoanabolic via stimulation of bone formation, which is mediated by Wnt signaling activation. Upon binding to the frizzled receptor and co-receptors, LRP5 and LRP6, Wnt activates a signaling pathway, leading to translocation of beta-catenin into the nucleus, specific gene expression, protein synthesis, and bone formation. Extracellular regulators of Wnt signaling include dickkopf 1 and sclerostin, a product of the *SOST* gene expressed by osteocytes that inhibits Wnt signaling [4,5]. PTH inhibits sclerostin and, therefore, stimulates bone formation.

Continuous exposure to PTH increases osteoclast activity, causing osteoporotic changes [6], mostly mediated by enhancing the production of RANKL (receptor activator of nuclear factor-κB ligand) and decreasing the production of osteoprotegerin (OPG), a natural decoy of RANKL, by osteoblasts and stromal cell. By binding to RANK (receptor activator of nuclear factor-κB), a member of the tumor necrosis factor family expressed by osteoclasts and their precursors, RANKL controls the differentiation, proliferation, and survival of osteoclasts [7]. As a result, continuous exposure to high levels of PTH causes bone loss, whereas intermittent exposure leads to bone mass gain.

## 2. CKD-Associated Secondary Hyperparathyroidism

Chronic kidney disease-mineral and bone disorder (CKD-MBD) involves a broad systemic disorder manifested in uremic patients by disturbances in mineral and bone metabolism and extraosseous calcification [8]. This syndrome comprises one or a combination of the following conditions: vascular or other soft tissue calcification, vitamin D deficiency, abnormalities in bone turnover, abnormal metabolism of calcium and phosphate, an increase of levels of fibroblast growth factor- 23 (FGF-23) and PTH.

**Figure 2:**
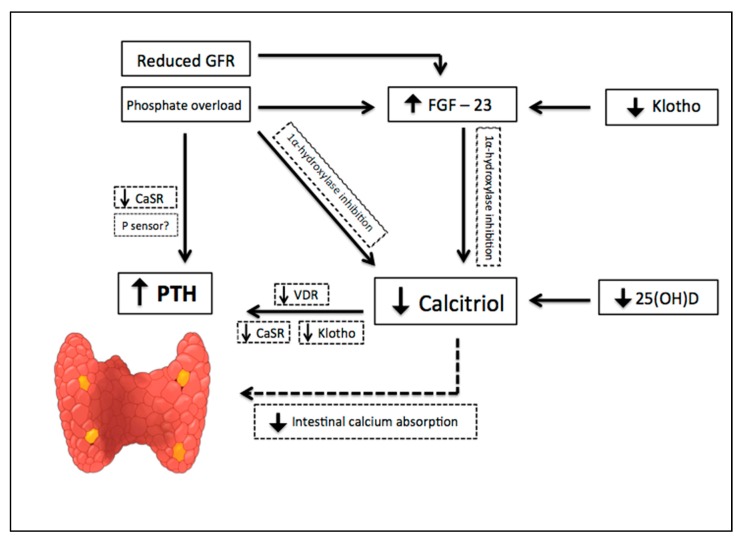
Pathogenic mechanisms of hyperparathyroidism progression in Chronic Kidney Disease (CKD). CKD progression is associated with phosphate overload, high levels of fibroblast growth factor- 23 (FGF-23), significant decrease in the expression of klotho, and a reduction of renal calcitriol production. Calcitriol deficiency influences parathyroid set point for calcium-regulated PTH secretion and decreases the expression of vitamin D and calcium receptors. Indirectly, calcitriol deficiency also stimulates PTH secretion due to a decrease in intestinal calcium absorption. Down arrow = decrease, Up arrow = increase.

The earliest abnormality that occurs with impaired kidney function is an increase in the level of FGF-23, a member of the family of the fibroblast growth factors which acts on phosphorus (P) metabolism. High FGF-23 results in increased phosphaturia, by inhibition of sodium-dependent P reabsorption (Na-P co-transporters IIa and IIc) [9], and deficiency of activated vitamin D, by inhibition of 1α hydroxylase [10]. For FGF-23 to exert its phosphaturic effect through FGF receptor, the klotho protein, expressed in the renal proximal tubules and parathyroid gland, is required as a cofactor. CKD progression is associated with a significant decrease in the expression of klotho, which causes high circulating levels of phosphate and vascular calcification in mice with CKD [11]. In addition, production of kidney calcitriol, the active form of vitamin D, decreases as CKD progresses. In normal conditions, calcitriol promotes intestinal absorption of calcium and phosphorus, and decreases the synthesis of PTH by binding to the vitamin D receptor (VDR) in the nucleus of the parathyroid cell. Therefore, calcitriol reduction allows an increase in the transcription of the PTH gene. Indirectly, it also stimulates PTH secretion due to a decrease in intestinal calcium absorption. Since parathyroid glands express FGF receptors and klotho [12], another mechanism regulating PTH secretion involves FGF-23, by reducing PTH mRNA through Klotho-dependent and Klotho-independent pathways [13]. However, as FGF-23 also inhibits the activity of 1α-hydroxylase, sustained high levels of FGF-23 are associated with an increase in PTH [10]. Calcitriol deficiency also influences the parathyroid set point for calcium-regulated PTH secretion and, possibly, decreases the expression of vitamin D and calcium receptors. Higher concentrations of calcium are needed to reduce PTH release in vitro from the parathyroid of uremic patients compared with healthy controls. Thus, renal klotho loss, hyperphosphatemia, vitamin D deficiency, and an increase in FGF-23 [12] are pathogenic mechanisms of hyperparathyroidism progression (Figure 2).

Secondary hyperparathyroidism (sHPT) is often observed in patients with CKD, mainly in those requiring dialysis therapy. PTH starts to rise when the estimated glomerular filtration rate (eGFR) drops to approximately 50 mL/min/1.73 m^2^. Further decline of renal function results in skeletal resistance to PTH, abnormal parathyroid growth and function. Persistent high levels of PTH generate an increase in FGF-23 expression and CKD osteodystrophy, favoring high bone turnover. This condition increases bone fragility, which may explain, at least in part, the association between sHPT and increased fracture risk. Furthermore, sHPT causes hyperphosphatemia, vascular and tissue calcification, anemia (by erythropoiesis impairment), and worse quality of life. The Dialysis Outcomes and Practice Patterns Study (DOPPS) has denoted that serum PTH higher than 600 pg/mL (63 pmol/L) is associated with a 21% increase in all-cause mortality risk [14].

Increased levels of FGF-23 and sHPT are closely related in the CKD setting. Whereas PTH acts directly on osteocytes to increase the FGF-23 expression, the FGF-23-receptor complex is downregulated in parathyroid glands, resulting in a loss of the ability of FGF-23 to decrease PTH expression. Moreover, as mentioned before, FGF-23 acts in the kidney by decreasing 1,25[OH]_2_D_3_ synthesis, and therefore contributing to enhance PTH levels.

Hyperphosphatemia has been recognized as an important player in the pathogenesis of sHPT [15]. Phosphate retention directly impairs renal 1α-hydroxylase activity, decreases 1,25[OH]_2_D_3_ synthesis, and affects posttranscriptional events that ultimately influence PTH mRNA stability and hormone synthesis. As CKD progresses, high phosphate levels influence the expression of factors involved in cell cycle regulation and parathyroid cell proliferation [16], and promote resistance to the actions of calcitriol in the parathyroid glands. High phosphate levels induce progression of sHPT and resistance to the effects of PTH in the bone. Moreover, it has been recently shown that phosphate at high concentrations acts as a noncompetitive antagonist of the CaSR, resulting in an increase of PTH secretion [17]. However, we should keep in mind that not only phosphate but other uremic toxins, such as indoxyl sulfate, are known to interfere with vitamin D metabolism and promote sHPT progression [18], as well as bone resistance to PTH.

Therapeutic arsenal for the treatment of sHPT includes calcitriol and vitamin D analogs, calcimimetics, and phosphate binders. Parathyroidectomy (PTX) is the surgical treatment indicated for cases with refractory hypercalcemia/hyperphosphatemia and severe symptoms such as extra skeletal calcification/calciphylaxis, intractable pruritus, and bone pain.

## 3. Effects of sHPT on BONE

Chronic PTH excess and bone resorption markers are associated with abnormal cortical and trabecular density, and fractures [19]. Cortical bone contributes to the mechanical strength of the skeleton, and this compartment is more adversely affected by hyperparathyroidism than the trabecular bone [20,21]. A histomorphometric evaluation in primary hyperparathyroidism has depicted that PTH promotes periosteal resorption and intracortical porosity [22].

A longitudinal study of 53 patients with stages 2 to 5 CKD, including those on dialysis, has shown that hyperparathyroidism and high serum concentration of bone turnover markers were associated with significant cortical loss, detected by dual-energy X-ray absorptiometry and high-resolution peripheral quantitative computed tomography [23]. The association of high levels of PTH and cortical porosity was also shown in dialysis patients [20]. In a post hoc analysis of the BRIC study, we observed an increased cortical porosity, evaluated through bone histomorphometry, which was associated with PTH levels, but not with the trabecular bone turnover [20].

In contrast to this effect in the cortical compartment, chronic excess of PTH might act as an anabolic agent for trabecular bone, increasing trabecular number and thickness [24]. Mice with prenatal conditional ablation of the PTH receptor (PTHR) by homologous recombination exhibit decreased trabecular bone compartment and increased thickness of cortical bone during fetal development [25].

Beyond consequences of PTH excess, the association between low levels of PTH and fractures is also a matter of debate. A U-shaped relationship between PTH and vertebral fractures has been shown in dialysis patients [26]. Moreover, Coco et al. [27] demonstrated a higher risk of hip fracture in patients on dialysis with lower PTH (around 195 pg/mL), and Iimori et al. [28] confirmed a high risk of fracture in patients with PTH levels below 150 pg/mL. However, according to Danese et al. [29], the risks for hip and vertebral fracture are weakly associated with PTH levels among patients on dialysis, with the lowest risk observed around a PTH concentration of 300 pg/mL.

Skeletal resistance to the PTH calcemic regulation further compromises the ability to maintain calcium levels in patients with advanced renal disease [30]. This resistance is not restricted to the effects of PTH on calcium release from the skeleton, but also involves the action of the hormone on the response of the bone cells [31]. We and others have shown increased bone expression of sclerostin in CKD patients [32,33], suggesting that this Wnt pathway inhibitor is related to the bone resistance to PTH. As a consequence, high serum PTH levels are needed to induce equivalent biologic responses in patients with CKD. Albeit some studies have described a down-regulation of PTHR in the context of CKD [34], we showed higher expression of PTHR1 in the osteocytes, mostly in earlier CKD stages [32]. However, this receptor activity was not evaluated in the mentioned study, and the current belief is that the PTHR activity might be compromised.

Some studies suggest that the transforming growth factor β (TGF-β) has a role in bone remodeling, regulating the recruitment of osteoclasts and osteoblasts. Downregulation of hormones and cytokines, such as IGF-1, IL-11, and TGF-β1, may be associated with age-related bone loss [35]. In vitro studies have shown that TGF-β increases the synthesis of bone proteins by osteoblastic cells [36], and local injection of TGF-β under the periosteum stimulates bone formation [37]. In contrast, transgenic mice overexpressing osteoblast-specific truncated TGF-β receptor present an increase in trabecular bone mass and a decrease in bone remodeling [38]. Furthermore, mice with knockout of the osteoblast TGF-β receptor develop a decrease in cortical bone and an increase in trabecular compartment, which is similar to the phenotype of animals expressing an active PTHR [39]. Thus, due to its dual effect, a fine balance of TGF-β production is required to prevent bone loss.

Pieces of evidence indicate that PTH and TGF-β operate together to exert their biological activities in the bone. Higher expression of TGF-β protein has been noted in high-turnover bones of patients with end-stage renal disease. In uremic animals, renal FGF-23 expression correlates with local TGF-β expression [40]. Patients with renal osteodystrophy have significantly higher levels of TGF-β than those without this condition [41]. Indeed, Santos et al. [42] demonstrated a significant high TGF-β expression in bone of patients with sHPT, which has improved after PTX.

PTH receptors are present in tissues unrelated to calcium homeostasis [43], but little is known about PTHR downregulation in the CKD context. Therefore, it is unknown whether PTH excessive levels, required to keep trabecular bone remodeling, might contribute to deleterious actions on nonclassical target organs.

## 4. Effects of sHPT on Cardiovascular System

sHPT promotes cardiovascular disease, regardless of calcium or phosphate levels [44]. There is some evidence of a correlation between serum levels of PTH and hypertension [45]. A study with 1784 individuals with mild renal dysfunction or normal eGFR followed for seven years revealed that PTH levels were able to predict hypertension in men, even after adjustments for age, smoking, and body mass index [46]. In addition, a meta-analysis with six prospective cohort studies, involving a total of 18,994 participants, showed that increased levels of PTH may be associated with a higher risk of hypertension [47]. Despite the evidence linking PTH with hypertension, the unanswered question is whether this relationship is causal. In particular, changes in systemic calcium metabolism are thought to play an important role in the regulation of blood pressure. Leiba et al. [48] have described some cases of hypertensive end-stage renal disease patients, who had a sudden drop in blood pressure after PTX. Heyliger et al. [49] documented a significant decrease in both systolic and diastolic blood pressure in 147 patients with hypertension undergoing PTX.

Parathyroid hyperfunction is associated with endothelial dysfunction and myocardial hypertrophy. In experimental studies using a rat model of CKD (5/6 nephrectomy) submitted to PTX, the continuous infusion of supraphysiological rates of 1–34 PTH was associated with myocardial hypertrophy and fibrosis along with a high myocardial expression of oxidative stress and inflammation markers [50]. There is an association between PTH levels, myocardial hypertrophy, and mortality, in patients with sHPT and in individuals from the general population [51]. An analysis with 2040 individuals has found that in women under 60 years and men over 59 years, PTH was a significant predictor of left ventricular hypertrophy [51].

PTH stimulation on cardiomyocytes promotes an increase in synthesis and expression of fetal-type proteins via activation of protein kinase C and affects contractile function by inhibiting β-adrenoceptor-mediated effects through the same pathway [52]. Therefore, this effect of PTH might suppress cardiomyocyte contractility.

Besides the aforementioned effect on cardiomyocytes, PTH acts on cardiac fibroblasts, inducing cardiac fibrosis in uremia [53]. As a promoter of fibroblast proliferation, TGF-β also generates cardiac fibrosis, as well as cardiomyocyte apoptosis and cardiac hypertrophy [54]. Beyond its role in bone turnover, PTH may mediate cardiovascular fibrosis and apoptosis through the TGF-β signaling pathway.

PTH might indirectly induce myocardial hypertrophy by increasing FGF-23 synthesis. High levels of FGF-23 have also been linked to left ventricular hypertrophy and mortality in patients with CKD. According to Gutierrez et al. [55], high levels of FGF-23 were independently associated with left ventricular hypertrophy in a group of 162 patients with CKD, not on dialysis. Furthermore, an experimental study has shown a direct effect of FGF-23 on myocardial hypertrophy [56].

CKD is associated with a high prevalence of arteriosclerosis or stiffening of the arteries independently of the presence of significant atherosclerosis. Calcification of the intimal and medial layers of vessels has an important role for arterial stiffening, and it is a key feature of the arterial disease in the kidney disease setting. Elevations in serum phosphate, calcium, and calcium-phosphate product, among other factors, are intimately involved in promoting calcification. Vascular calcification has been frequently documented in primary hyperparathyroidism and sHPT, although its pathophysiology is still a matter of debate. The question is whether there is a direct deleterious effect of PTH on vessels. An experimental study has shown that exposure of vascular endothelial cells to PTH led to decreased expression of the mRNA of OPG, a known protection factor of endothelial tissue [57]. However, the administration of 1–34 PTH to young mice has attenuated the progression of aortic valve calcification in a model of CKD [58]. Therefore, the association of long-term exposure of high PTH levels with vascular and valvular calcification [59] is probably explained by the large supply of calcium and phosphate from high bone turnover [60]. Indeed, sHPT is associated with an increased risk of calcific uremic arteriolopathy, also known as calciphylaxis. Elevations in phosphate and calcium levels compromise the vasculature, resulting in ischemic changes and plaque-like lesions that progress to painful skin lesions [61]. The prognosis of this condition is poor, and patients’ survival generally reaches one year, in the best scenario [62].

In summary, although the role of PTH in mediating the RANK/RANKL/OPG axis in skeletal and extraskeletal calcification [63] has not yet been elucidated, this hormone should be included in the list of mediators of the bone–vascular interaction.

## 5. Effects of sHPT on CKD Progression

A recent meta-analysis suggests an independent association between serum phosphate levels, kidney failure, and mortality among patients with CKD not requiring dialysis [64]. Increased levels of phosphate may lead to tubular injury, interstitial fibrosis, endothelial dysfunction, and vascular calcification via phosphate or calcium-phosphate crystals [65].

A retrospective cohort study with 13,772 incident patients on hemodialysis has revealed an association between the decline of residual kidney function and abnormalities of MBD, such as high levels of phosphate, intact PTH, alkaline phosphatase, and low levels of calcium [66]. It has also been shown that diabetic predialysis CKD patients with sHPT usually require greater healthcare resource utilization and experience a faster kidney disease progression with a higher risk of dialysis initiation or death when compared with predialysis diabetic patients with CKD but without sHPT [67].

## 6. Effects of sHPT on CKD-Related Caquexia and Energy Expenditure

Muscle weakness is another condition possibly associated with hyperparathyroidism in patients with CKD, whose physiologic basis is not completely understood. Some studies using muscle biopsies of patients with CKD have revealed a decrease in muscle mitochondrial oxidative enzymes, such as citrate synthase and cytochrome c oxidase, and a decrease in the synthesis of contractile muscle proteins, myosin heavy chain, and mitochondrial proteins [68].

The diagnosis of uremic myopathy is based on clinical features and a multifactorial origin, caused by physical inactivity, reduced protein intake, immunological and myocellular alterations, inflammation, metabolic acidosis, abnormalities in insulin-like growth factor, and myostatin expression. Most of these mechanisms stimulate the ATP-dependent ubiquitin-proteosome system (UPS), one of the most important intracellular proteolysis pathways [69]. In addition, vitamin D deficiency is also recognized as a risk factor for CKD-related myopathy [70].

Experimental evidence supports the toxic effect of PTH on muscles. Animals exposed to high doses of PTH for four days showed muscle dysfunction, including a reduction of mitochondrial activity and a decrease in high-energy phosphate [71]. Gomez-Fernandez et al. [72] have described the weakness of the respiratory muscles in correlation with PTH levels.

Weight loss, a common feature of advanced CKD, might be related to the excess of PTH levels. CKD patients with sHPT who undergo PTX present, in general, a significant magnitude of weight gain (more than 5% above the baseline) [73]. Interestingly, patients undergoing dialysis with sHPT present an increase of resting energy expenditure that reduces significantly six months after PTX [74].

Kir et al. [75] uncovered evidence that PTH and PTH-related protein (PTHrP) signal, through the same receptor, work as potential mediators of body weight loss, in association with browning of adipose tissue and loss of muscle mass. They observed that injection of cancer cells into mice was responsible for increased concentration of PTHrP, with capacity to activate the uncoupling protein-1, inducing “browning” of white adipose tissue and energy generation. The pathway to browning includes PTH/PTHrP activation of protein kinase A and loss of muscle mass via the UPS. Deletion of PTHR abrogates the muscle atrophy and changes the regulation of thermogenic genes in mice with 5/6 nephrectomy.

This finding revealed that the deletion of PTHR in animal models acts as a critical factor for resistance to the development of sarcopenia. A higher concentration of brown adipose tissue might be an important factor associated with muscle wasting in CKD by increasing energy expenditure.

## 7. Effect of sHPT on Glucose Metabolism

Glucose intolerance is another condition associated with uremia, possibly due to decreased insulin secretion. Some evidence suggests that PTH has a role in this event since insulin resistance has been noted in patients with primary hyperparathyroidism [76]. There is, however, no convincing evidence of such effect in uremia.

Experimental studies in uremic animals have demonstrated an action of PTH on protein kinase C promoting an increase of cytosolic calcium in pancreatic islets. Excess PTH may interfere with the ability of the beta-cells to augment insulin secretion appropriately, affecting insulin secretion by calcium-dependent mechanisms [77]. Ahamed et al. [78] have documented a negative correlation between PTH and fasting insulin in uremic patients. Patients with severe hyperparathyroidism had relatively more impairment of pancreatic beta-cell function in comparison with those with mild hyperparathyroidism, and an intravenous dose of 1-cholecalciferol has been associated with an improvement of beta-cell function.

Mak et al. [79] have shown that patients on dialysis with 1,25-(OH)_2_D_3_ deficiency and sHPT were glucose-intolerant and insulin-resistant. After intravenous administration of 1,25-(OH)_2_D_3_, there was an increase of insulin secretion and an improvement of glucose tolerance. These events occurred without any change in serum PTH concentration. An improvement of glucose tolerance and insulin secretion has been described in children with uremia after handling sHPT markers, by phosphate restriction and oral phosphate binders [80]. Therefore, changes in metabolism could be explained by a reduction of PTH, phosphate and/or FGF-23 levels, as well as by normalization of serum calcitriol.

However, some studies have shown that the undercarboxylated form of osteocalcin, an osteoblast-specific protein, is associated with energy metabolism regulation [81]. Infusion of undercarboxylated osteocalcin improves glucose tolerance and insulin resistance in mice with insulin receptor deletion [82]. In sHPT, there is a PTH-mediated increase of carboxylated and undercarboxylated osteocalcin, which might lead to an increase in insulin sensitivity and energy expenditure [83]. Thus, these conflicting actions of PTH on glucose metabolism should be addressed in future studies.

## 8. Effect of sHPT on Central Nervous System

Toxic effects of PTH on the central nervous system have been suggested. The mechanism might involve an increase of levels of cytosolic calcium in brain synaptic terminals since the removal of parathyroid is capable to prevent an excess of calcium in uremic brains [84]. Guisado et al. [85] have shown a correlation between changes in the electroencephalogram (EEG), similar to those presented in the chronic uremia context, with the increase of brain calcium content. Furthermore, PTX before uremia induction prevented EEG abnormalities, whereas the administration of parathyroid extracts to healthy animals induced EEG changes similar to those observed in uremic animals.

It has also been suggested a harmful effect of PTH on cognitive function in CKD patients. However, there is still weak support due to the lack of research in this area [86].

## 9. Effect of sHPT on Hematopoietic and Immunological System

Hyperparathyroidism aggravates hematopoietic dysfunction [87], inhibiting erythropoiesis, accelerating erythrocyte sedimentation rate, and increasing osmotic fragility of erythrocytes through Ca-ATPase stimulation [88]. Elevated levels of PTH cause fibrosis of bone marrow [89], related to the synergism between FGF and TGF-β action on myofibroblast transdifferentiation. A regression of medullary fibrosis was demonstrated one year after PTX in uremic patients, accompanied by a reduction in IL-1, TNF-a, TGF-β, and FGF [42].

Cell-mediated immunity, involving lymphocyte function, is abnormal in uremia [90]. Chronic exposure to PTH is associated with a reduction of T lymphocyte proliferation, cytokine production, and impairment of immunoglobulins production. There are some beneficial effects of PTX on the immunologic parameters in the sHPT context. In this regard, patients with CKD on maintenance dialysis followed prospectively for one year after PTX presented an improvement in serum immunoglobulins and complement titles. This improvement in humoral immunity after PTX occurs probably due to the remarkable reduction of PTH, which directly affects B-cells, and partially improved nutritional state [91]. Thus, abnormalities of the ‘‘uremic immunodeficiency’’ may be related to the degree of sHPT.

Interestingly, T lymphocytes are essential to the PTH-mediated bone homeostasis. Some authors have shown the role of T lymphocytes in PTH–mediated skeleton homeostasis, suggesting that the immune system is essential to the bone actions of PTH. Hory et al. [92] have reported that transplantation of human parathyroid into athymic mice did not promote bone resorption. A subsequent study by Pettway et al. [93] has suggested an involvement between T cells and bone response to PTH. Intermittent PTH treatment could induce a faint anabolic response in the trabecular bone compartment of T cell-deficient mice.

## 10. Conclusions

sHPT has significant clinical implications not restricted to the pathophysiology of some mineral and bone metabolism disorder. There is a substantial body of evidence that supports the role of PTH in the pathogenesis of abnormalities in cell function, contributing to several uremic findings in patients with CKD by increasing intracellular calcium. The presence of PTH receptors in different tissues unrelated to calcium homeostasis may be the reason for such a number of nonclassical effects of severe sHPT.

**Figure 3:**
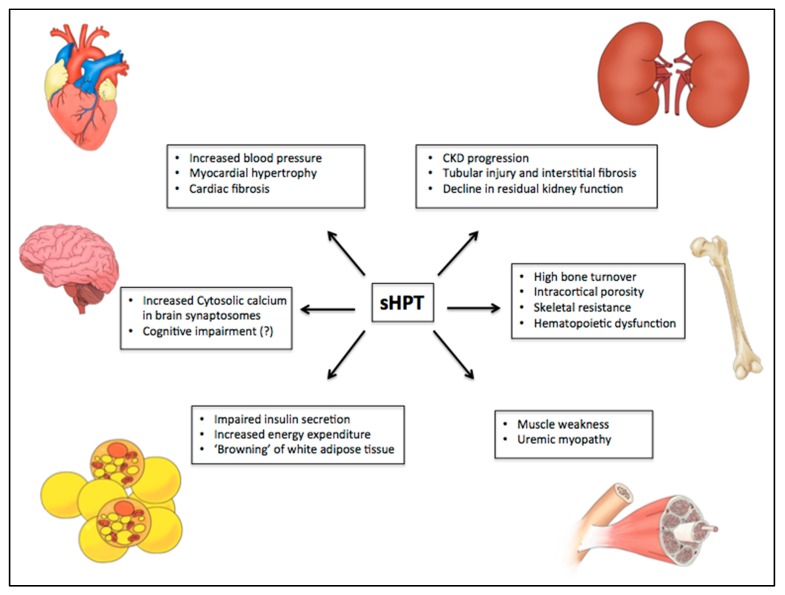
PTH-related manifestations on different target organs in uremic syndrome. Some studies have suggested the role of PTH in uremic syndrome through calcium-dependent and independent mechanisms. Among several toxic actions, it has been shown an association of high levels of PTH with myocardial hypertrophy and cardiovascular disease, nervous system disorders, development of sarcopenia, progression of chronic kidney disease, hematopoietic dysfunction, reduced insulin secretion by pancreatic beta-cells, increase of energy expenditure, “browning” of white adipose tissue, and high bone turnover with significant cortical compartment loss. CKD: chronic kidney disease.

The diversity of toxic effects involves myocardial dysfunction, cardiac hypertrophy, muscle weakness, osmotic fragility of erythrocytes, glucose intolerance, and abnormalities of the immune system (Figure 3). Moreover, there is evidence that PTX restores some of these organ dysfunctions, as shown in Table 1.

Therefore, in view of the several adverse effects of high levels of PTH, a true uremic toxin, there is a need for closer monitoring for its levels in patients with CKD, in order to achieve more rigorous and early control of sHPT.

## Figures and Tables

**Table 1:** PTH-related manifestations and beneficial effects of Parathyroidectomy.

PTH-Related Manifestations	Parathyroidectomy Effects	Ref.
Hypertension Myocardial hypertrophy	Blood pressure reduction Beneficial effect on cardiovascular mortality	[44,46]
Abnormal bone density (mainly in cortical compartment)	Improvement of bone mineral density at lumbar spine and femoral neck	[19,94]
Increase of levels of cytosolic calcium in brain synaptic terminals	Improvement of cognitive function Prevention of electroencephalogram abnormalities in uremic animals	[85,95]
Hematopoietic dysfunction Accelerate erythrocyte sedimentation rate Increase of osmotic fragility of erythrocytes	Improvement of anemia Regression of medullar fibrosis	[87,96]
Reduction of T lymphocyte proliferation and cytokine production Impairment of immunoglobulins production	Improvement on serum immunoglobulins and complement titles	[90,91]
Increase in all-cause mortality risk	Improve of survival in patients with severe secondary Hyperparathyroidism Improvement of quality of life	[14,97]
